# Numerical Analysis of 2-D Positioned, Indoor, Fuzzy-Logic, Autonomous Navigation System Based on Chromaticity and Frequency-Component Analysis of LED Light

**DOI:** 10.3390/s21134345

**Published:** 2021-06-25

**Authors:** Jae-Hoon Jeong, Kiwon Park

**Affiliations:** 1 The School of IT, Information and Control Engineering, Kunsan National University, Gunsan-si 54150, Korea; jh7129@kunsan.ac.kr; 2 School of Mechanical and Automotive Engineering, Youngsan University, Yangsan-si 50510, Korea

**Keywords:** vehicle-navigation system, GPS-shaded areas, vehicle-positioning system, chromaticity

## Abstract

Topics concerning autonomous navigation, especially those related to positioning systems, have recently attracted increased research attention. The commonly available global positioning system (GPS) is unable to determine the positions of vehicles in GPS-shaded regions. To address this concern, this paper presents a fuzzy-logic system capable of determining the position of a moving robot in a GPS-shaded indoor environment by analyzing the chromaticity and frequency-component ratio of LED lights installed under the ceiling. The proposed system’s performance was analyzed by performing a MATLAB simulation of an indoor environment with obstacles. During the simulation, the mobile robot utilized a fuzzy autonomous navigation system with behavioral rules to approach targets successfully in a variety of indoor environments without colliding with obstacles. The robot utilized the x and y coordinates of the fuzzy positioning system. The results obtained in this study confirm the suitability of the proposed method for use in applications involving autonomous navigation of vehicles in areas with poor GPS-signal reception, such as in tunnels.

## 1. Introduction

Many studies have been conducted to develop autonomous navigation systems that can facilitate safe and efficient mobility of vehicles [1,2,3,4,5]. Autonomous navigation systems combine new technologies, such as modern sensors, information communication, and intelligent control, to enable a vehicle to recognize its surrounding environment, analyze risks, and achieve active safety [1,2,3,4,5]. Furthermore, the development of autonomous navigation technology could enhance the safety of driving via improvements in the range of recognition and reaction time, reduction in road accidents, alleviation of traffic congestion, and promotion of the automotive convergence industry [1,2,3,4,5].

Behavior-based navigation algorithms are commonly used for developing autonomous navigation systems [6,7,8,9]. They determine the motion of a vehicle by using programmed behavior rules corresponding to the real-time information of the surroundings obtained from the mounted sensors. Moreover, these algorithms improve navigation skills, such as ensuring coping ability for unexpected situations while heading to a target and avoiding obstacles. However, to achieve more advanced navigation, multiple behavior rules generating a variety of vehicular motions corresponding to surrounding information must be included, and the problems involving combination between behavior rules should be considered [6,7,8,9].

Fuzzy-logic theory mimics human recognition ability, and it is widely employed in studies on autonomous navigation because it exhibits good performance in processing information gathered from the surrounding environment [6,7,8,9]. Fuzzy logic includes three stages: fuzzification, rule evaluation, and defuzzification. In the fuzzification process, all sensor data input to the fuzzy system have their own weights assigned by input membership functions. In the rule-evaluation process, the behavior rules are activated for sensor data with nonzero weights. In the defuzzification process, all the outputs from the behavior rules are combined using the defuzzification function. Because all the weights of the input signals are considered, and the behavior rules are well combined to produce an output—such as the motion of a robot. Fuzzy logic demonstrates excellent performance in systems addressing multiple environmental uncertainties, such as robot navigation algorithms [6,7,8,9].

Positioning is one of the most important techniques in autonomous navigation [1,2,3,4,5]. A variety of positioning methods based on sensor techniques are currently utilized in the autonomous navigation systems of vehicles. Further, studies are underway to minimize the measurement errors that occur for each sensor and enhance the accuracy of the positioning results by utilizing the advantages of each positioning sensor [1,2,3,4,5].

Commonly used positioning sensors present advantages as well as disadvantages. In the case of the global navigation satellite system (GNSS), the position of the receiver is calculated by using satellite networks through triangulation, utilizing the time at which the satellite signal arrives at the ground-surface receiver and the position information of other satellites. However, owing to various error factors, a positioning error of approximately 7 m occurs [10,11,12,13,14,15]. In addition, differential GNSS uses the ground master station to address satellite clock, ion/ionospheric, and orbit errors; the positioning error is approximately 2 m in this case, and obtaining an accurate measurement is still difficult [1,2]. Some studies have combined inertial navigation systems (INS), vision, radar, geomagnetic sensors, Wi-Fi, etc., to improve accuracy. However, Wi-Fi has a different positioning accuracy depending on the interval of installation and the number of surrounding signal sources (access points, APs). In INS-GPS, owing to the sensor bias and noise accumulation over time, the accuracy and reliability of positioning decrease [10,11,12,13,14,15]. In addition, GPS, which is widely used for vehicle positioning, measures a vehicle’s current position through a satellite using a function for transmitting navigation signals; consequently, it cannot measure the vehicle’s position in indoor and other GPS-shaded areas [3]. Therefore, INS-GPS cannot produce precise position measurements in areas where signal reception is difficult. Thus, while studies are continuously being conducted on sensor-measurement fusion technologies, many issues must still be addressed [10,11,12,13,14,15].

To improve positioning in GPS-shaded areas, many researchers have begun focusing on the visible light communication (VLC) technique using light emitting diode (LED) lighting [10,11,12,13,14,15]. VLC requires multiple LED networks, in which a unique ID is assigned to each LED transmitter, and positions are calculated via trilateration, whereby the distances from a receiver to the three closest transmitters are measured. Various measurement methods for LED-based VLC are available, including proximity [16], finger printing [17], received signal strength [18], angle of arrival [19], time of arrival, time difference of arrival, phase difference of arrival, and image-based positioning [20]. Although the proximity has a simple and inexpensive process, it yields low accuracy [21]. In contrast, the finger printing, received signal strength, arrival data (i.e., angle of arrival, time of arrival, time and phase difference of arrival), and image-based techniques are well-known for achieving high accuracy in LED-based indoor positioning. However, the equipment size and the complexity of hardware hinder their practical application [22,23].

In our previous study, a fuzzy-logic-based positioning system was developed using the chromaticity and frequency components of LED light [24], and it could successfully detect the position of a mobile robot in the developed simulator.

In this present study, the fuzzy positioning system was implemented for two autonomous mobile robot navigation systems, and the performance of the fuzzy positioning system in each navigation system were compared. The first navigation system comprises fuzzy-logic–based behavior rules that determine the motion of the mobile robot while it moves toward a target and avoids obstacles simultaneously. The second navigation system is based on the potential field method wherein the robot finds a path such that the attractive force from the target is maximized and the repulsive forces from obstacles are minimized [25,26]. The mobile robot operating based on the two proposed navigation algorithms utilizes the chromaticity and frequency component ratio of LED light obtained via the optimal route to avoid and escape obstacles and reach targets without having any pre-installed localized information.

## 2. Positioning Method

### 2.1. Experimental Environment

**Figure 1:**
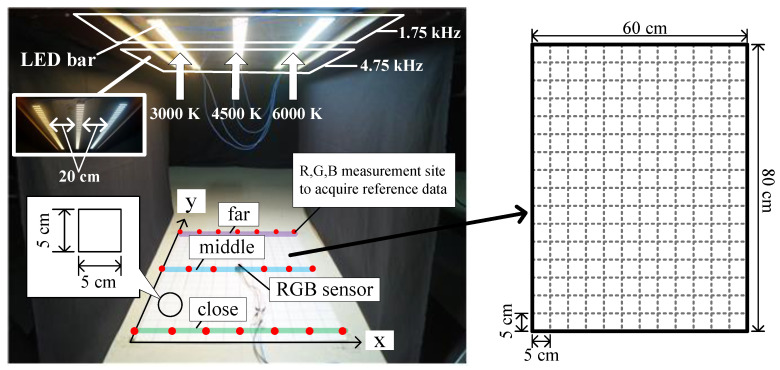
Indoor-environment simulator used in this study and size of testbed [24].

In this study, we developed a simulator that imitates GPS-shaded areas, as depicted in Figure 1. The color temperatures of LED illuminators depicted in Figure 1 equaled 3000, 4500, and 6000 K, and the said illuminators were installed 73 cm above the simulator floor. Each LED-lighting fixture measured 1 m long—a combination of two 50 cm long bar lamps. Individual fixtures were placed 20 cm apart. The simulator floor was marked by a grid comprising square elements measuring 5 cm each along the x- and y- directions, as depicted in Figure 1. On the grid, the red dots indicate points of intersections between the horizontal and vertical grid lines, and they represent chromaticity measurement points. Therefore, in this study, reference-data measurements were performed at 21 points.

It is noteworthy that the front and rear bar lamps comprising the LED illuminator operated at different frequencies, thereby separating the illumination zones along the y-axis. Figure 1 shows the irradiation of 1.75 kHz light on the elliptical part of the LED illuminators and that of 4.75 kHz light on the rectangular part.

By configuring the simulator in this manner, the positioning in the x-axis direction is calculated based on the chromaticity value, whereas that in the y-axis direction is calculated based on the different frequency-component ratios of the LED-irradiated light.

**Figure 2:**
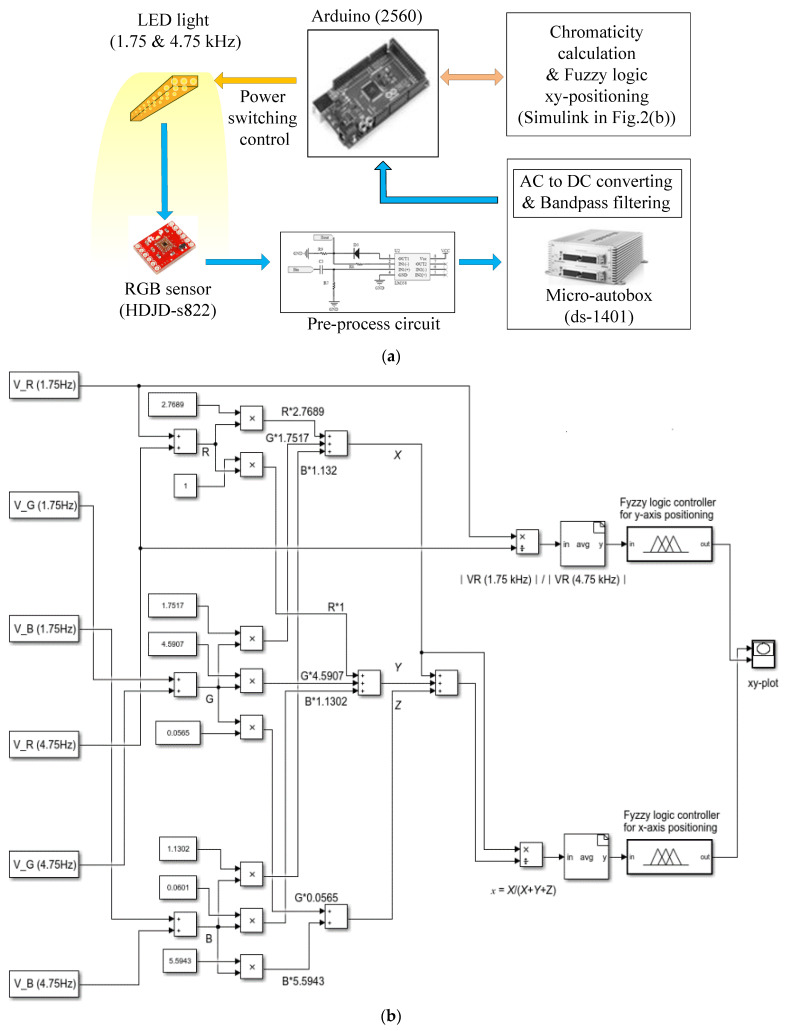
Signal-processing workflow: (**a**) Schematic of signal-processing procedure; (**b**) Simulink model for fuzzy positioning.

Figure 2 shows the signal-processing procedure used in this study [24]. An RGB sensor (HDJD-s822) is used for performing positioning on the mesh grid on the simulator floor. The RGB sensor detects the light irradiated by the three LED fixtures located on the ceiling of the simulator and outputs R, G, and B voltage signals. The LED illuminators irradiate 1.75 and 4.75 kHz light for each section, as shown in Figure 1. For this purpose, the LED illuminators are controlled using a power switching method that employs pulse width modulation.

To block the effects of other external lights and to select only the driving frequency components of the LED illuminators, the preprocessed signals were passed through a bandpass filter that allowed only the 1.75-kHz and 4.75-kHz signals to pass. The Direct-Form-II infinite-impulse-response bandpass filter was designed using MATLAB FDA Toolbox and implemented using MATLAB Simulink. The Simulink model of the bandpass filter works with the Micro-Autobox (ds-1401) DAQ to conduct signal filtering. The DAQ output signals were converted into DC via a smoothing circuit comprising a capacitor, following which the chromaticity value and frequency-component ratio were calculated using Arduino 2506. Finally, the x- and y-axis coordinates were estimated using the corresponding fuzzy systems. Processing in the Arduino board was implemented as a MATLAB Simulink model linked to the board via the MATLAB Embedded Coder Toolbox.

### 2.2. Fuzzy Positioning System

In the constructed simulator, three LEDs with different colored temperatures in the x-axis direction were installed on the ceiling. The overlapping of light was considered, following which different chromaticity values were measured depending on the position of the RGB sensor in the x-axis direction.

**Figure 3:**
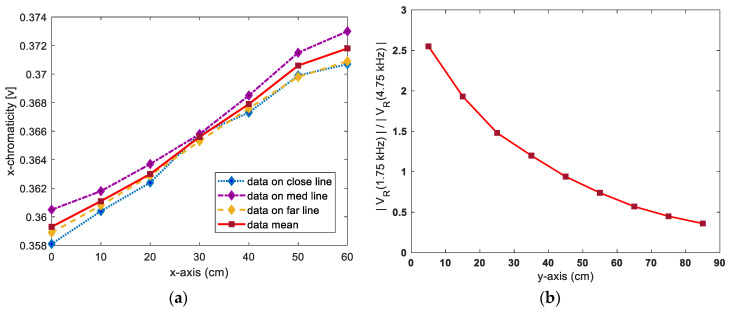
Reference-data variations along x- and y- axes: (**a**) Chromaticity variation along x-axis; (**b**) frequency-component-ratio variation along y-axis.

We first collected reference chromaticity data corresponding to the positional coordinates in the x-axis direction. The red dot on the mesh grid in Figure 1 denotes the location of the collected reference data. Thus, for obtaining reliable measurements of the x-axis in the interior of the simulator, the x coordinate was increased from zero to 60 cm in increments of 10 cm, and the chromaticity data were collected at each point on the lines indicated by “close”, “med” and “far” intersecting each x-axis coordinate. Figure 3a shows the variation in the mean values of chromaticity data along the x-axis.

As shown in Figure 1, the bar-shaped LED illuminators installed on the ceiling of the tunnel simulator were driven by power-switching methods at 1.75 and 4.75 kHz for each section. In this study, the y-axis position coordinate was calculated using the 1.75 kHz frequency-component ratios included in the sensor output signal, corresponding to the position change along the y-axis.

**Figure 4:**
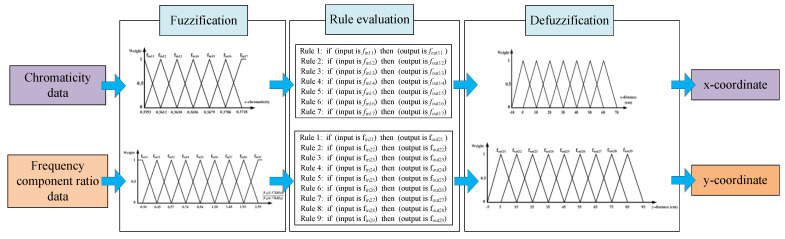
Schematic of fuzzy positioning system [24].

For collection of the reference data, the RGB sensor was fixed at 30 cm on the x-axis. As the sensor was moved along the y-axis from 5 to 85 cm in increments of 10 cm, the magnitudes of the 1.75 and 4.75 kHz signals, subject to the bandpass filter and smoothing circuit, were measured. These measurement values were obtained to calculate the frequency-component ratios (|VR(1.75 kHz)|/|VR(4.75 kHz)|). Figure 3b shows the frequency-component ratios corresponding to the y-axis coordinates at which the sensor was located. Based on the reference data shown in Figure 3, a fuzzy positioning system was developed [24]. In this study, a fuzzy-logic positioning system was designed with MATLAB to calculate the x and y-coordinates corresponding to the position of a mobile robot from data pertaining to the chromaticity and the frequency-component ratio of the light. Data points corresponding to x-chromaticity and the frequency-component ratio (i.e., points other than those shown in Figure 3) were estimated by using linear approximation between the two-boundary data, which involved every two-neighbored reference data point in Figure 3. In this way, all the data points corresponding to the x-chromaticity and frequency-component ratio at the location of the mobile robot in the MATLAB-based simulation were estimated; these were then utilized as inputs to the fuzzy positioning system which calculates the 2-D Cartesian coordinates. Figure 4 summarizes the fuzzy positioning process [24].

## 3. Fuzzy-Logic Autonomous Navigation System

### 3.1. Sensors of Navigation System

**Figure 5:**
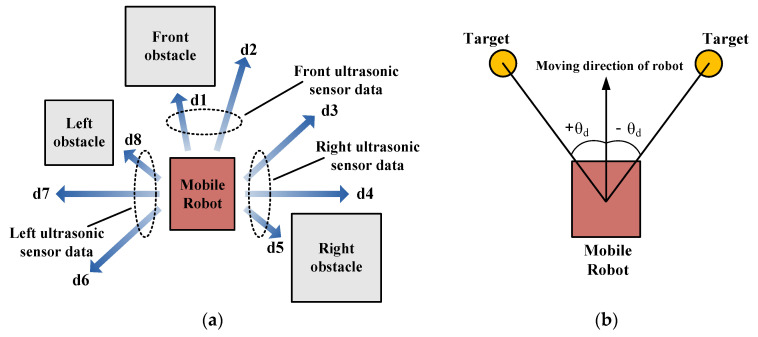
Performance of sensor systems of mobile robot: (**a**) description of distance sensors; (**b**) description of positioning system.

Figure 5 describes the sensor system of the mobile robot used for evaluating the performance of the developed navigation system. The mobile robot was mounted with eight distance sensors and the fuzzy positioning system investigated in [24]. Figure 5a shows the function of the distance sensors mounted at the front, right, and left of the robot. Among the distance values, three minimum values from each side, expressed as fd = MIN {d1, d2}, rd = MIN {d3, d4, d5}, ld = MIN {d6, d7, d8}, were inputs to the fuzzy navigation system. Figure 5b shows the function of the fuzzy positioning system that uses the x and y coordinates of the robot’s current position to calculate the angle (θd) between the current moving direction and the direction to the target. θ_d_ is also used as an input to the fuzzy navigation system.

To reach the target, the mobile robot requires navigation skills, such as heading to the target, avoiding obstacles, and following the edges of obstacles. The effectiveness of these skills has been verified in previous studies [10,11,12,13,14,15].

### 3.2. Fuzzification Process of Distance Data

**Figure 6:**
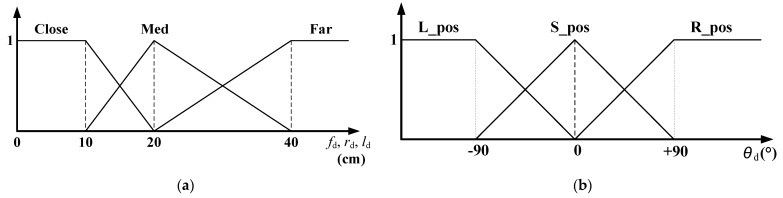
Membership functions for the fuzzification of input data: (**a**) membership function for the fuzzification of distance data; (**b**) membership function for the fuzzification of positioning data.

During the fuzzification process, each input (f_d_, r_d_, l_d_, and θ_d_) to the fuzzy navigation algorithm can be classified as close, med, or far and subsequently assigned weight values between zero and one using the fuzzy membership functions. Figure 6a depicts the membership function used for the fuzzification of inputs f_d_, r_d_, and l_d_. As can be realized, the figure depicts weight values linearly corresponding to the distance data between each grid. Accordingly, the fuzzy navigation system facilitates the mobile robot to perform a linear motion based on its distance from obstacles. Because grid points corresponding to named sub-functions within each membership function are considered as criteria for determining the robot’s distance from obstacles as well as its direction of motion and speed via sensor signals, any variations in the grid directly affect the motion of the mobile robot. For example, a reduced range of the “close function” in Figure 6a causes the robot to approach the obstacles better. This is due to the narrowing of the recognition range for an input to be classified as “close”.

The sensitivity of the observed variations in assigned weight values is related to the slope of the triangular membership function. In other words, a steep (high) slope implies that the robot would respond more actively to its distance from obstacles. As depicted in Figure 6a, the slope of the triangular function between grid points 10 and 20 is steeper compared with that in other regions, and this makes the robot to avoid obstacles more quickly in that region. Accordingly, the input θ_d_ is classified as either L_pos, S_pos, or R_pos, and a weight value between zero and one is assigned to each variable based on the membership function, as described in Figure 6b. In this study, the grid points within input membership functions were adjusted to determine the optimum path to the target.

### 3.3. Behavior Rule-Evaluation Process

In the rule-evaluation process, the behavior rules consisted of “if–then” statements, such as “if(l_d_ and f_d_ and r_d_ and θ_d_) then (LVel and RVel).” LVel and RVel are the output values of each behavior rule and are used to generate the real control signal of the speed of the two wheels. In this study, 81 behavior rules, consisting of navigation skills, movement towards the target, following of obstacle edges, and avoiding of obstacles, were designed for the stable motion control of the robot during navigation. Table 1 lists some of the behavior rules to make the robot move toward the target. Table 2 lists some behavior rules to make robot follow the edge of obstacles. Table 3 lists some behavior rules for the robot to avoid obstacles. In the rule-evaluation process, only rules with all non-zero weights for the input variables, f_d_, r_d_, l_d_, and θ_d_ were activated because the output of each behavior rule is the minimum weight value among the input variables.

### 3.4. Defuzzification Process to Produce Robot Motion

**Figure 7:**
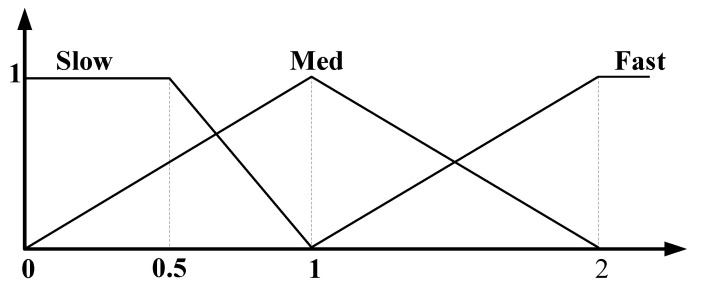
Output membership function.

The outputs of multiple activated rules in the rule-evaluation process were combined to produce the control signals for the two wheels of the robot. This was done by using the output membership function in Figure 7 and the weighted average method given in Equation (1).

(1) $$x=\frac{\sum_{i=1}^{number\;of\;activated\;rules}\left(m^{i}\times w^{i}\right)}{\sum_{i=1}^{number\;of\;activated\;rules}m^{i}}\;$$

In Equation (1), *x* is the defuzzified control signal for the speed of the two wheels, and m is the output from the activated rules, i.e., the minimum non-zero weight of activated rules. *w* is the center value; 0.5, 1, and 2 correspond to “Slow”, “Med” and “Fast” functions, respectively, in the output membership function in Figure 7. Because the center values in the output membership function affect the speed of the mobile robot, the center values are selected to generate the optimal path.

## 4. Potential Field Autonomous Navigation System

### 4.1. Sensors of the Navigation System

When the robot that is based on the potential field navigation technique reaches the destination, it is affected by the attractive force from the goal position and the repulsive forces from obstacles.

**Figure 8:**
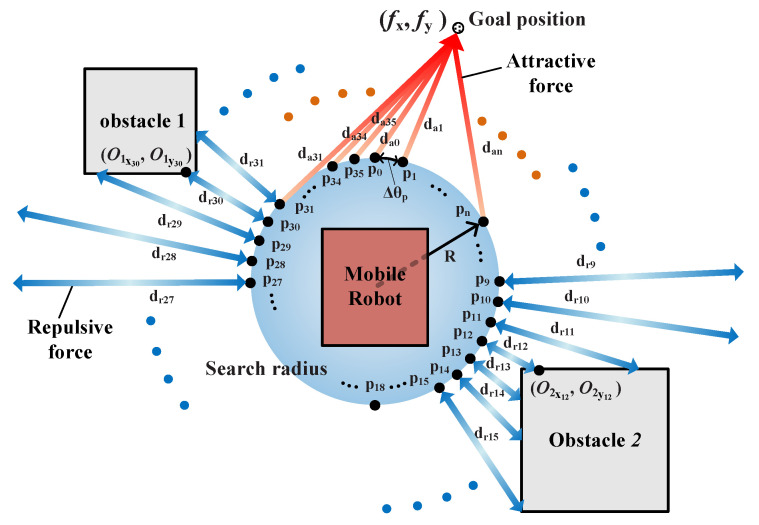
Sensor system of the potential field navigation system (search radius).

Figure 8 presents the sensor system of the potential field–based mobile robot navigation system. To calculate the influences of the forces and finding path on the goal position, a virtual circle surrounding the mobile robot is designed, where the attractive and repulsive forces are calculated on the points of the circle, in the interval of ∆*θp* from 0 to 360° as indicated by p0 − p35 in Figure 8. The coordinate of the search radius is expressed as follows:

(2) $$p_{n_{x}}=x\left(k\right)+R_{x}\cos\left(n\;∆\theta_{p}\right),\;\;p_{n_{y}}=y\left(k\right)+R_{x}\sin\left(n\;∆\theta_{p}\right)$$

where {*x*(*k*), *y*(*k*)} is the coordinate of the current position of the mobile robot, and $∆\theta_{p}=2\pi /m$; therefore, *n* = 0, …, *m* − 1, and R is the radius centered on {*x*(*k*),*y*(*k*)}, which is the position of the robot.

The detection range of the sensor system for the surrounding obstacles is spread in a manner similar to the spokes of a wheel with the center of the search radius as the hub. Further, all the distances between the points on the search radius and the obstacles are calculated to obtain the repulsive forces from the points on the search radius, which are depicted as d_r0_ − d_r35_ in Figure 8. In addition, the sensor system calculates all the distances between the points on the search radius and the goal position to evaluate the attractive forces from the points on the search radius, which are depicted as d_a0_ − d_a35_ in Figure 8. When the robot is heading toward the target, 72 datasets comprising d_r0_ − d_r35_ and d_a0_ − d_a35_ are used in every step to calculate the steering angle of the mobile robot in the next step using the potential field navigation algorithm.

### 4.2. Potential Field Navigation Algorithm

The potential field algorithm uses the two virtual forces (repulsive and attractive forces) to lead the robot to the destination. The repulsive force interacts between the robot and obstacles and pushes the robot away from the obstacles. The attractive force interacts between the robot and the goal position to pull the robot toward the destination. Both forces are calculated using the distance data from the 36 points on the searching radius surrounding the robot, to the obstacles and goal positions, respectively.

The attractive force is a positive value, which is proportional to the distance from the goal position, and it becomes “0” at the goal position. The attractive force from $p_{n}$(nth point on the search radius) in Figure 8 is expressed as follows:

(3) $$U_{n_{\;attractive}}\left(p_{n}\right)=C_{a\;}\left[{\left(f_{x}-p_{n_{x}}\right)}^{2}+{\left(f_{y}-p_{n_{y}}\right)}^{2}\right],$$

where $C_{a\;}$ is the coefficient used for balancing the effect of the attractive force, ($f_{x}$, $f_{y}$) are the coordinates of the goal position, and ($p_{n_{x}}$, $p_{n_{y}}$) is the x and y coordinates of point $p_{n}$.

The repulsive force is inversely proportional to the distance from an obstacle, and it approaches “0” as the robot moves farther from the obstacle. The repulsive force from $p_{n}$ on the search radius corresponding to the *i*^th^ obstacle is described in Figure 8 and expressed as follows:

(4) $$U_{n_{\;repulsive}}\left(p_{n}\right)=\frac{C_{r}}{{\left(f_{x}-O_{i_{x_{n}}}\right)}^{2}+{\left(f_{y}-O_{i_{y_{n}}}\right)}^{2}},$$

where $C_{r\;}$ is the coefficient used for balancing the effect of the repulsive force.

Therefore, the total potential ($U_{n{\;}_{total}}$) from $d_{n}$(n^th^ distance data) in the search radius described in Figure 8 is expressed as the summation of the attractive force ($U_{n{\;}_{attractive}}$) and repulsive force ($U_{n{\;}_{repulsive}}$), as given below:

(5) $$U_{n{\;}_{total}}=U_{n_{\;attractive}}+U_{n_{\;repulsive}}.$$

After calculating *n* potential data, the steering angle of the mobile robot in the next step (θ(k+1)) is determined as $∆\theta_{p}n_{min}$, where $n_{min}=\left\{n|\min\left(U_{n_{total}}\right)\right\}$ to move the robot to the position where the value of the total potential ($U_{n{\;}_{total}}$) is minimized.

## 5. Design of Autonomous Navigation Simulator

**Figure 9:**
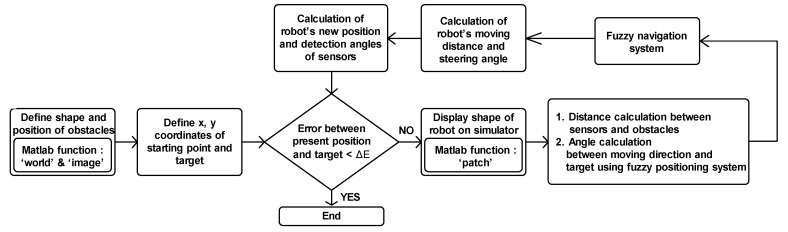
Flowchart of data processing in robot simulator.

In this study, a mobile-robot navigation simulator was developed using MATLAB, and it was applied for the verification of the autonomous navigation performance of the robot. The navigation environment was developed using the MATLAB function “world” in the matrix form with the same size as the floor area of the indoor environmental simulator (Figure 1), which was 60 cm × 80 cm. The size of the matrix was 600 × 800, which implies that the interval between elements was 1 mm, and each element had a color value; for example, “1” for white and “0.5” for grey. In addition, the MATLAB function “image” was used to visualize the navigation environment consisting of a white background with grey obstacles. The motion of the robot was indicated as an overlapped rectangle by using the MATLAB function “patch”, corresponding to the calculated moving direction and the speeds of the two wheels. Figure 9 depicts the sequence of the data-processing operation performed in the navigation simulator. Because the x and y coordinates, representing the robot’s position, are calculated by using the speeds of the two wheels, the denser path in the simulator indicates a slower speed. The eight distance sensors that produce the input values (d1–d8) for the fuzzification process do so by calculating the distances between the location of each sensor and obstacles placed in front of the heading direction of the sensors.

**Figure 10:**
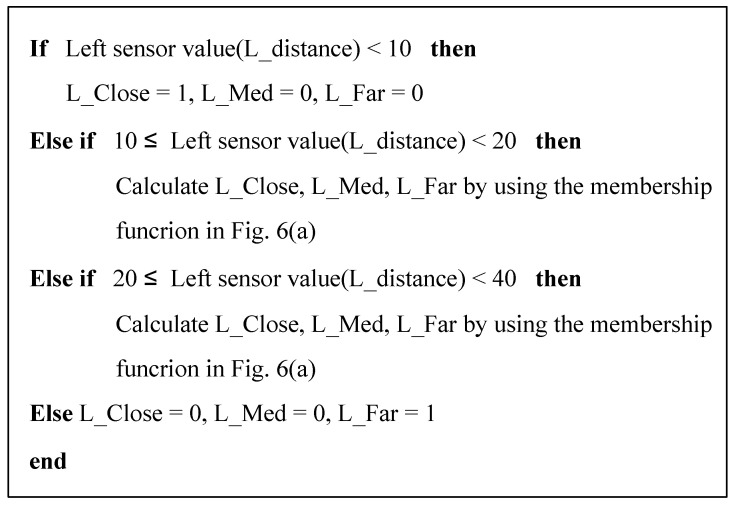
Sensor data fuzzification algorithm.

Figure 10 shows the algorithm of the MATLAB code for fuzzification of the left sensor data in the proposed navigation simulator. The figure describes the code for the input membership function illustrated in Figure 6a, and it can be used to generate 12 fuzzified variables—L, F, R_Close, L, F, R_Med, L, F, R_Far, L, S, and R_pos—from the front, right, and left sensor signals. The fuzzification of θd calculated using the output of the fuzzy positioning system was performed using the MATLAB code for the input membership function, as illustrated in Figure 6b.

**Figure 11:**
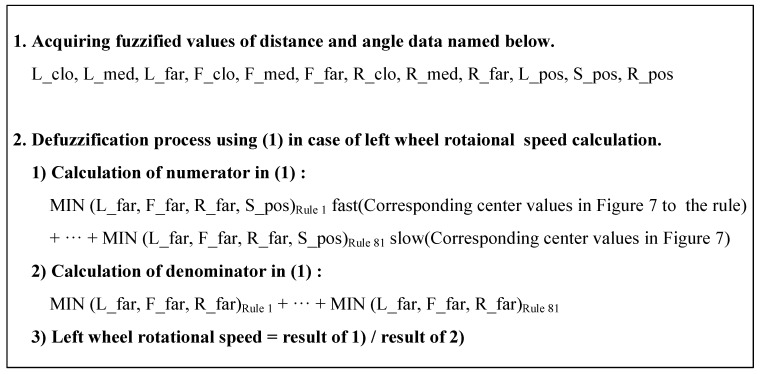
Rule evaluation and defuzzification algorithm.

Figure 11 shows the algorithm of the MATLAB code for the rule evaluation and defuzzification algorithm (based on (1)) developed as part of the proposed navigation simulator.

**Figure 12:**
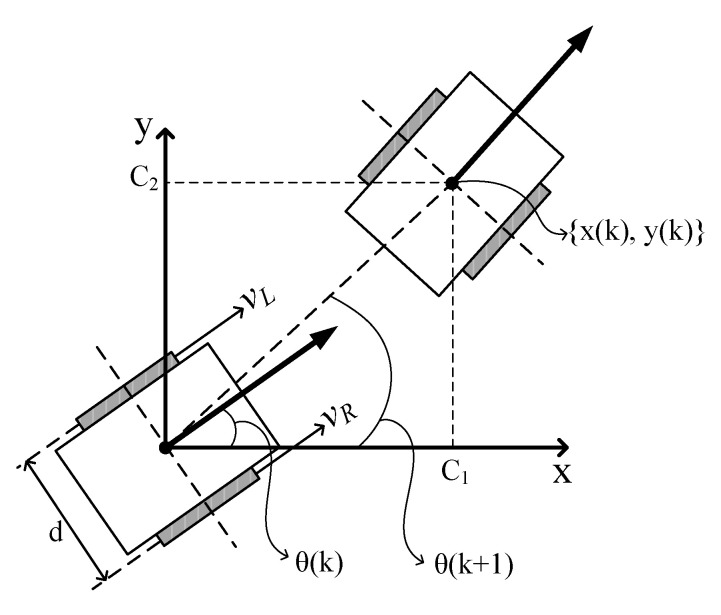
Parameters influencing simulation of robot motion.

The behavior rule evaluation and defuzzificaton procedures are used to calculate the rotational speed (LVel, RVel) at the center of the wheels of the mobile robot. The outputs from the fuzzy navigation system are expressed as follows:

(6) $$v_{L}=LVel\;K\;r=\omega_{L}\;r,$$

(7) $$v_{R}=RVel\;K\;r=\omega_{R}\;r$$

where *K* (rad/s) is the coupling constant between the outputs (*LVel*, *RVel*) from the fuzzy navigation system and the real rotational velocity (*v_L_*, *v_R_*) of the wheels, and r is the radius of the wheel. Finally, the renewal position coordinate of the robot, as shown in Figure 12, is calculated using (8) and (9), where T is the sampling interval that was set to 0.33 s in this study.

(8) $$\theta \left(k+1\right)=\theta \left(k\right)+\left(\frac{V_{R}-V_{L}}{d}\right)T$$

(9) $$\left\{\begin{array}{c} x\left(k\right)\\ y\left(k\right) \end{array}\right\}=\sqrt{C_{1}^{2}+C_{2}^{2}}\;\left\{\begin{array}{c} \cos\theta \left(k+1\right)\\ \sin\theta \left(k+1\right) \end{array}\right\}$$

While the fuzzy logic navigation approach calculates the steering angle and moving distance in the next step based on the velocity difference between the two wheels of the mobile robot, as shown in (8) and (9), the potential field navigation approach only calculates the steering angle for steering the robot in the direction where the value of the total potential is minimized. Hence, the moving distance of the potential field approach was calculated with a function that linearly relates the moving distance of the robot in each step to the minimum value among 36 distance data.

## 6. Results

**Figure 13:**
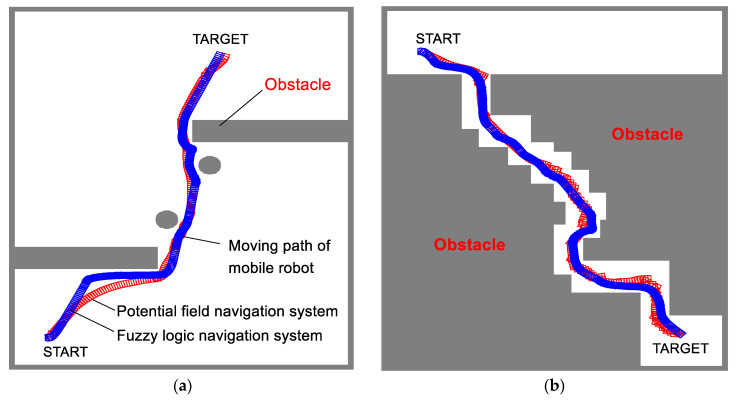

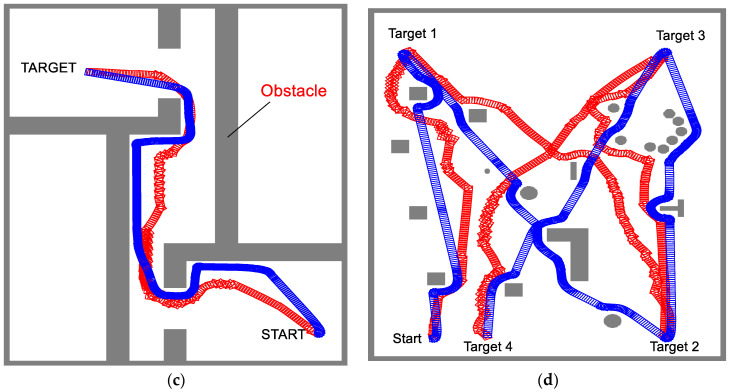
Simulation results of autonomous fuzzy navigation system with fuzzy positioning—(**□**: navigation path using fuzzy logic navigation system, **□**: navigation path using potential field navigation system): (**a**) navigation path to reach target after avoiding obstacles; (**b**) navigation path in narrow pathway; (**c**) navigation path to reach to the target in a room; (**d**) navigation path to reach multiple targets.

In this study, the performances of two navigation systems (fuzzy logic and potential field navigation systems, both utilizing the fuzzy positioning system) were evaluated with the developed robot navigation simulator. Figure 13a–d show the simulation results, paths of the robot, and avoidance of obstacles until the robot reaches the targets in various environments. In the results, the robot in each moving step is symbolized by square shapes, wherein the blue and red colors represent the moving path of the robot using fuzzy logic and potential field navigation, respectively. In the moving path, the denser parts of overlapped square shapes indicate that the moving velocity is lesser than that of the other parts.

Figure 13a shows the robot’s path to the target after it avoided round and square-shaped obstacles; the navigation is shown to be successful without any collisions occurring with the obstacles. As shown in Figure 13b, the robot navigated through a passageway to reach the target, and Figure 13c shows the path from one room to another. Figure 13d shows the performance of the navigation system in an environment with scattered obstacles. The robot reached the final target after three other targets without any collisions.

Table 4 summarizes the simulation results of the study in terms of path length, navigation time, and robot velocity. For the fuzzy navigation system, the velocity of the robot varies depending on the value of the coupling constant K in Equations (6) and (7). In this study, the value of K was set such that it minimized the errors between the simulation results and the performance of the actual robot, the latter being subject to a function of hardware specifications. Ultimately, the values of K and r were set to 1 rad/s and 3 cm, respectively. For the potential field navigation system wherein the linear approximation method was used to calculate moving distance in each step, the linear function was set such that it produced distance values in the range from 3–4 cm corresponding to the minimum distance value in the simulation environment, and these results indicate that the potential field navigation system shows a higher moving velocity than the fuzzy logic navigation system.

For an obstacle, the behavioral rules for following the edge of the obstacles in the fuzzy logic navigation system were activated in the fuzzy rule evaluation stage; the robot with fuzzy logic navigation follows the edge of obstacles, which results in a higher path length (Figure 13a), whereas the robot with potential field navigation moves toward the middle of the pathway because the repulsive potential is minimized here, resulting in a shorter path length (Figure 13b–d). Thus, the moving paths of the two navigation techniques are different.

Among all results, the fuzzy logic navigation system showed a smoother moving path than the potential field navigation system, as shown in Figure 12. The 81 behavior rules as well as the 4 input and 2 output membership functions in the fuzzy logic navigation system interact well; further, the fuzzy logic navigation system produced smoother moving paths than the potential field navigation system, wherein the steering angle is calculated according to the repulsive and attractive potential data.

A faster navigation velocity was observed in simulation 4 than in other simulations while simulation 4 also exhibited longer navigation time and distance. We think that the navigation velocity was affected by the density of surrounding obstacles, resulting in a faster moving velocity in the environment with scattered obstacles (simulation 4) compared with the other environments.

According to the results, the fuzzy positioning process in [24] demonstrated good performance because the chromaticity and frequency component data utilized during the simulation were gathered within the simulator environment (Figure 1) via the bandpass filtering, without the interference of other lights. However, for real-time applications of the proposed technique, an improved filter technique is required, along with an investigation of various chromaticity and frequency component applications of LED light, to minimize the influence of other lights that cause positioning errors.

## 7. Conclusions

In this study, an autonomous robot navigation system was developed for indoor environments using the fuzzy logic navigation system and potential field navigation system, and both systems utilized the fuzzy positioning system. The positioning system utilized chromaticity and frequency component ratio data, which were obtained from the LED lighting that was installed under the ceiling in the simulation environment.

To evaluate the performance of the autonomous navigation system, various simulations that mimicked indoor environments were conducted. The navigation simulator generated the path of a mobile robot using the proposed systems until the robot reached the target. When the robot navigated by the fuzzy logic navigation system through the unknown environment, it used eight virtual distance sensors, and the steering angle and moving distance were calculated using 81 behavior rules and fuzzy membership functions. When the robot navigated by the potential field navigation system, the repulsive and attractive forces on 36 points of the searching radius, which is a circle surrounding the mobile robot, were used to calculate steering angle. This also led the robot to the position of minimum potential. The moving distance in the potential field navigation system was calculated with a linear function that produces the moving distance corresponding to the minimum distance data to surrounding obstacles in the following step.

The x and y coordinates of both navigation systems were calculated using the fuzzy positioning system based on pre-measured chromaticity and frequency component ratio data. The robot successfully reached the targets in various simulation environments using both navigation systems.

In this study, the possibility of implementing LED lighting–based indoor navigation systems was evaluated. In the future, a practical test will be performed using practical robots.

## Figures and Tables

**Table 1:** Behavior rules for target steering motion in navigation.

Rule	if	ld	and	fd	and	rd	and	θd	then	LVel	and	RVel
1	if	Far	and	Far	and	Far	and	S_pos	then	Fast	and	Fast
2		Far		Far		Far		L_pos		Fast		Slow
3		Far		Far		Far		R_pos		Slow		Fast

**Table 2:** Behavior rules for following edge of obstacles motion in navigation.

Rule	if	ld	and	fd	and	rd	and	θd	then	LVel	and	RVel
4	if	Far	and	Far	and	Close	and	L_pos	then	Med	and	Med
5		Close		Far		Far		R_pos		Med		Med

**Table 3:** Behavior rules for avoiding obstacles motion in navigation.

Rule	if	ld	and	fd	and	rd	and	θd	then	LVel	and	RVel
6	if	Med	and	Close	and	Close	and	any	then	Slow	and	Fast
7		Close		Close		Med		any		Fast		Slow
8		Close		Med		Close		any		Med		Med

**Table 4:** Rules for avoiding obstacles in robot navigation.

Simulation Data		Simulation 1 (Figure 13a)	Simulation 2 (Figure 13b)	Simulation 3 (Figure 13c)	Simulation 4 (Figure 13d)
Length of path	Fuzzy logic navigation	79.60 cm	88.41 cm	111.86 cm	299.78 cm
	Potential field navigation	77.36 cm	105.11 cm	128.90 cm	333.40 cm
Navigation time	Fuzzy logic navigation	99.34 s	133.33 s	161.39 s	312.21 s
	Potential field navigation	57.43 s	76.24 s	92.74 s	223.10 s
Mean of velocity	Fuzzy logic navigation	0.80 cm/s	0.66 cm/s	0.69 cm/s	0.96 cm/s
	Potential field navigation	1.35 cm/s	1.38 cm/s	1.39 cm/s	1.49 cm/s
